# The role of small extracellular vesicles in spreading and inhibiting arthropod-borne diseases

**DOI:** 10.3205/dgkh000503

**Published:** 2024-10-23

**Authors:** Iman Owliaee, Mehran Khaledian, Ali Shojaeian, Armin Khaghani Boroujeni

**Affiliations:** 1 Department of Medical Virology, Faculty of Medicine, Hamadan University of Medical Sciences, Hamadan, Iran; 2 Department of Medical Entomology, Faculty of Medicine, Hamadan, Iran; 3 Research Center for Molecular Medicine, Institute of Cancer, Avicenna Health Research Institute (AHRI), Hamadan University of Medical Sciences, Hamadan, Iran; 4 Skin Disease and Leishmaniasis Research Center, Isfahan University of Medical Sciences, Isfahan, Iran

**Keywords:** small extracellular vesicles, arthropod-borne diseases, arbovirus-borne diseases, exosomes, flaviviruses

## Abstract

Arthropod-borne diseases (ABDs) refer to a group of viral pathogens that affect a wide range of vertebrate hosts, including humans and non-human primates. In addition to being transmitted by mosquitoes and ticks, arthropods can also spread pathogens that cause severe human diseases. On the other hand, extracellular vesicles (EVs) can serve as cross-placental drug delivery vehicles (DDVs) to the fetus and even as antigen-presenting cells (APCs). To this end, the current review aimed to examine the role of small EVs (sEVs) in the transmission and inhibition of arthropod-borne viruses, also known as arboviruses. First, a deeper understanding of the mechanistic aspects of how these vesicles function during insect-pathogen interactions is required. Next, scalability and yield optimization must be addressed while introducing EV-based therapeutics on an industrial scale in order to implement them effectively. Finally,it is recommended to consider that sEV-mediated transfer plays a crucial role in the spread of ABDs. This is because it transfers pathogenic agents between cells within vectors, resulting in subsequent transmission to hosts. Consequently, sEVs provide potential targets for the development of novel therapies that inhibit pathogen replication or reduce arthropod vector populations. Future research in this area should emphasize how these vesicles function within host-vector systems, using advanced imaging techniques – such as high-resolution microscopy (HRM) – and cost-effective methods, in order to produce sufficient quantities for large-scale implementation.

## Abstract (de)

Arthropoden-übertragbare Krankheiten (ABK) beziehen sich auf eine Gruppe von viralen Krankheitserregern, die ein breites Spektrum von Wirbeltieren einschließlich Menschen und nicht-menschlichen Primaten befallen. Neben der Übertragung durch Stechmücken und Zecken können Arthropoden auch Erreger verbreiten, die beim Menschen schwere Krankheiten verursachen. Andererseits können extrazelluläre Vesikel (EV) als Vehikel zur Übertragung von Arzneimitteln über die Plazenta auf den Fötus und sogar als Antigen-präsentierende Zellen (APCs) dienen. Daher wurde in der vorliegenden Übersichtsarbeit die Rolle kleiner EVs (kEV) bei der Übertragung und Hemmung von durch Arthropoden übertragenen Viren, bekannt als Arboviren, untersucht. Zunächst ist ein tieferes Verständnis der mechanistischen Aspekte der Funktionsweise dieser Vesikel während der Interaktionen zwischen Insekten und Krankheitserregern erforderlich. Als Nächstes müssen die Skalierbarkeit und die Optimierung der Ausbeute bei der Einführung von EV-basierten Therapeutika in industriellem Maßstab angegangen werden, um sie effektiv einsetzen zu können. Schließlich sollte berücksichtigt werden, dass die kEV-vermittelte Übertragung eine entscheidende Rolle bei der Verbreitung von ABK spielt, weil Pathogene zwischen Zellen innerhalb von Vektoren übertragen werden, was zu einer anschließenden Übertragung auf den Wirt führt. Folglich stellen kEV potenzielle Ziele für die Entwicklung neuer Therapien dar, die die Replikation von Krankheitserregern hemmen oder die Populationen von Arthropodenvektoren reduzieren. Künftige Forschungsarbeiten in diesem Bereich sollten sich auf die Funktionsweise dieser Vesikel in Wirts-Vektor-Systemen konzentrieren und dabei fortschrittliche bildgebende Verfahren wie die hochauflösende Mikroskopie und kosteneffiziente Methoden einsetzen, um ausreichende Mengen für eine groß angelegte Umsetzung zu produzieren.

## 1 Introduction

### 1.1 Arthropod-borne diseases (ABDs) and small extracellular vesicles (sEVs)

Main arthropod vectors such as mosquitoes are responsible for transmitting a wide range of pathogens, including flaviviruses, that cause severe human diseases [1]. In 1882, Carlos Juan Finlay theorized that *Aedes aegypti* (formerly known as *Culex cubensis*) transmitted yellow fever; however, his theory was not proven. Almost two decades later, in 1901, Walter Reed confirmed that the Aedes mosquito was indeed responsible for transmitting yellow fever. Subsequently, Max Theiler et al. [2] isolated the virus and repeatedly passed it through various tissue culture techniques, leading to the creation of a highly effective, attenuated yellow-fever vaccine strain 17D, for which they were awarded the Nobel Prize in Physiology or Medicine.

The largest risks facing blood transfusion safety originate from the relationship between certain mosquito-borne arboviruses and explosive epidemic outbreaks, increasing the likelihood that the majority or all of the 130+ arboviruses known to cause human disease are transmitted through blood transfusions [3]. The *Ixodes scapularis* tick is a hematophagous parasite that feeds on blood and carries many positive-stranded ribonucleic acid (RNA+) viruses [4], [5]. These viruses, which belong to the Flaviviridae family, pose a significant threat to human health worldwide. The family is divided into four genera: flavivirus, hepacivirus, pegivirus, and pestivirus. The latest classification published by the International Committee on Taxonomy of Viruses (ICTV) identifies 89 species within these four genera [2]. Flaviviruses, such as the dengue virus (DENV), Zika virus (ZIKV), tick-borne encephalitis virus (TBEV), West Nile virus (WNV), Powassan virus (POWV), and Langat virus (LGTV), are transmitted by mosquitoes or ticks and can cause a range of symptoms, including paralysis, fever, meningitis, shock, congenital anomalies, and even death [4], [6]. Once such viruses reach epidemic proportions, they pose a grave threat to public health [7], [8].

**Figure 1:**
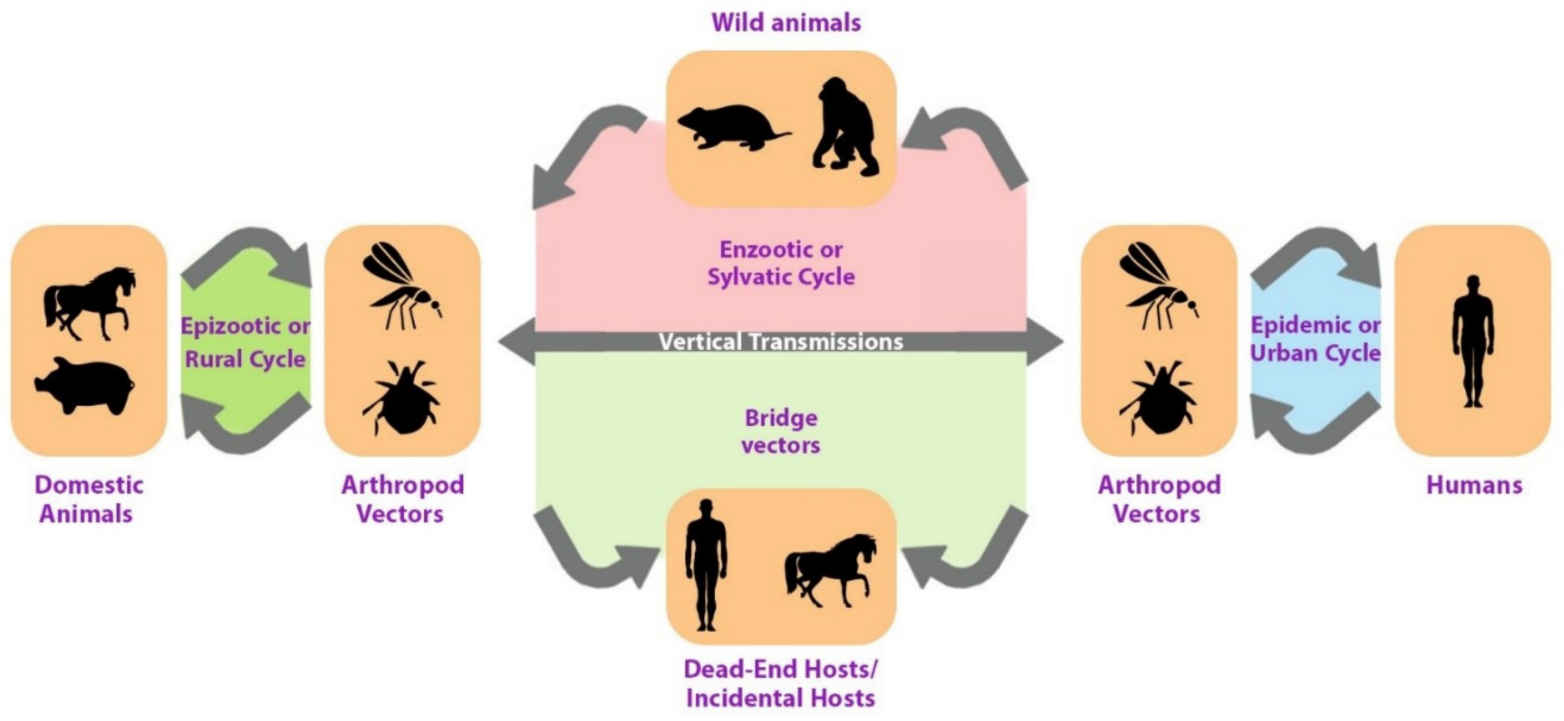
Simplified arbovirus transmission cycle

In addition, these viruses require a host, typically a bird or a small mammal, to replicate alongside a vector, such as a mosquito, in order to spread to other organisms. Therefore, female mosquitoes consume the virus from the blood of an infected animal, which is then transmitted to the new host via the mosquito’s saliva when it bites another animal. Thus, the most common arbovirus hosts are birds, humans, and other mammals, such as horses (Figure 1 (Fig. 1)), which are typically dead-end hosts because they cannot serve as mosquito reservoirs. Notably, however, clinical illnesses can result from the infection of nonviable hosts [9].

It has been assumed that the most effective method for developing treatments and vaccine candidates is to place a strong emphasis on the essential vector molecules utilized by flaviviruses to transmit to their vertebrate hosts [1]. Due to the absence of vaccines and antiviral drugs for the vast majority of viruses, mosquito or vector control remains essential for reducing the risk of disease or virus transmission [8], [10]. Notable difficulties exist in eradicating the pathogen-transmitting mosquito species *Aedes aegypti* and *Aedes albopictus*, as Aedes can reproduce rapidly indoors or outdoors in small bodies of water and find their native habitat in densely populated tropical cities [8].

Given that RNA viruses have emerged or re-emerged over the past few decades, there have been an increased number of epidemics with rapid geographical spread. In this regard, both pathogenic flaviviruses are primarily transmitted by *Aedes* spp. mosquitoes [7]. Numerous viruses, including some flaviviruses, have been discovered to exploit the biogenesis pathways of EVs to secrete specific viral RNAs and proteins or even to aid virus assembly and egress [11]. Given this perspective, the purpose of this review is to investigate how EVs affect the transmission or inhibition of arboviruses, as they are of utmost importance in this context. Viruses, such as the Japanese encephalitis virus (JEV), rotavirus, and the human immunodeficiency virus (HIV), frequently exploit the cellular biogenesis machinery and the EV secretion for virion construction and release. Other viruses, such as the human cytomegalovirus (hCMV) and the Epstein-Barr virus (EBV), also use EVs to evade the immune system and spread viral regulatory elements, such as proteins, RNAs, and microRNAs (miRNAs), from an infected cell to its neighbors [12], [13], [14].

Limited research indicates that Exosomes produced by flavivirus-infected cells play a role in viral infection and transmission.They have also been implicated in the transmission of DENV serotype 2 (DENV2) from arthropods to mammalian cells, mediated by interactions with either the Chikungunya virus (CHIKV) intracellular replication by the host endosomal sorting complex required for transport (ESCRT) [15] or through the augmentation of antibody-mediated CHIKV infection, which has demonstrated infection development and the worsening of disease severity [16].

Recent research has investigated the role of sEVs in ABD transmission and inhibition. sEVs are nanoscale membrane-bound vesicles that play important roles in intercellular communication by transporting bioactive molecules between cells. Consequently, the role of sEVs in spreading and inhibiting ABDs is discussed in the current paper.

As illustrated in Figure 1 (Fig. 1), the transmission of arboviruses involves two ecologically and genetically distinct cycles, namely the enzootic or sylvatic cycle and the human-amplified or urban cycle. Therefore, arboviruses maintain their existence in nature by cycling between a host, an organism that carries the virus, and a vector, an organism that carries and transmits the virus to other organisms. Thus, the host can be either a vertebrate or an invertebrate. Vectors are typically arthropods, such as mosquitoes and ticks. Additionally, the virus replicates in both host and vector. Once forest-infected humans enter urban environments, arbovirus-based infections can rapidly spread through transmission by highly anthropophilic urban mosquitoes. It is then said that the sylvatic transmission cycle has bled into the urban transmission cycle. 

### 1.2 Small extracellular vesicles

Exosomes are unique among extracellular vesicles (EVs) due to their distinct size and origin from other vesicles [17]. Thus, apoptotic bodies (ABs), microvesicles (MVs), and exosomes are examples of EVs that are frequently classified according to their size and biological origin. MVs are created by budding at the cell surface and are estimated to be between 100 and 1,000 nm in size. ABs are also referred to as enormous vesicles released by apoptotic cells. In addition, exosomes are generated by endocytic processes within the multivesicular body (MVB), and their size ranges from 40 to 150 nm. In addition to these primary groups of vesicles, it is believed that additional subpopulations of MVs and exosomes with distinct cargos and functions exist. Notably, EVs are a variety of communication tools released by cells in both healthy and pathological states [18], [19], [20]. Once detected by a receptor cell, the small extracellular vesicles (sEVs) secreted by various cells into bodily fluids such as blood, saliva, urine, and breast milk can regulate a vast array of biological processes [21].

Even the blood-brain barrier (BBB) and the placenta have been discovered to be permeable to EVs. The composition of sEVs also varies according to the type of cell they originate from or the cellular state of the cell [18], [22], [23]. Reportedly, sEVs can transmit some enveloped and non-enveloped viruses, which is distinct from virion-mediated infection. Historically, virologists have studied these sEVs as non-infectious or flawed particles. Exosomes have been described as carrying RNA, miRNA, proteins, or other molecules that modulate cell communication, thereby inducing changes in the target cells during physiological processes and viral infections [18]. This is intriguing because the budding process of EVs is similar to that of viruses, allowing RNA viruses to use EVs to transfer viral particles or even naked viral genomes to infectious host cells [11].

In addition, exosomes contain a higher concentration of cholesterol, an essential component in the replication of flaviviruses. Exosomes released from flavivirus-infected host and vector cells have revealed highly distinct populations with molecular repertoires that dictate their intracellular communication, viral propagation, and host immune system functions [17], [19]. Furthermore, viruses have frequently utilized the EV biogenesis pathway to facilitate viral infection and spread [24]. Recent research has demonstrated that arthropod EVs can facilitate flavivirus transmission and that flavivirus infection modifies the EV cargo, although the role of mosquito EVs in viral infection remains unknown [25].

Moreover, studies have similarly revealed that certain diseases use exosomes to control their environments by transporting a variety of bacteria. Exosomes, for instance, have been described as a mode of communication utilized by the malaria-causing parasite *Plasmodium falciparum* [4]. Viruses and EV biogenesis pathways have a large number of established connections. Human immunodeficiency virus (HIV), the herpes simplex virus 1 (HSV-1), the Ebola virus, and the rabies virus have previously been reported to capture the ESCRT pathway for assembly and egress. In addition, it is believed that the tetraspanin CD63 is utilized by human herpesvirus 6 during replication (HHV-6). Additionally, other flaviviruses, including DENV, can recruit specific host ESCRT subunits to replication sites [18].

EVs are also associated with pathological conditions such as thrombosis, cancer and viral infections such as HCV, HIV, and DENV. Notably, dengue patients have more CD41+ EVs in their bloodstream and lymph stream system than control groups, and the DENV causes platelets to release EVs in the laboratory. In addition, some EVs released from DENV-activated platelets contain interleukin-1 (IL-1), which, when combined with tumor necrosis factor-alpha (TNF-α), can become a significant factor in dengue inflammation, plasma leakage, and endothelial activation. In addition, the number of EVs in the plasma of dengue patients correlates with the severity of the disease. Consequently, EVs can be utilized to understand the pathophysiology of dengue hemorrhagic fever (DHF) [26].

Exosomes have recently garnered medical attention for their significance in the study of autoimmune and cancer diseases, and they are now recognized as potentially novel therapeutic targets for neurological disorders, such as Parkinson’s disease. In the past decade, the discovery of their innumerable distinct functions has provided them with additional potential biological relevance [2], [4]. Due to their recent involvement in pathophysiological processes such as cancer, neurological disorders, cardiovascular diseases (CVDs), metabolic syndrome (MetS), and even fibrosis, exosomes are a topic of particular interest today [7], [27]. Therefore, EVs can be used as potential biomarkers for diagnosing a variety of diseases and have therapeutic potential.

Selecting a suitable technique for isolating pure EVs appears crucial for research and therapeutic analyses [16]. In general, it has been demonstrated that EVs regulate interactions between the host and the pathogen in both directions. These vesicles further disseminate their infectious content to promote or inhibit infections. Pathogen-related substances, such as viral RNA or proteins, the pathogen’s immunological escape, and the suppression of the host immune system are all means by which pathogenic interests are promoted [28].

EVs contain the RNA genomes of the ZIKV, the porcine reproductive and respiratory syndrome (PRRS) virus, and the WNV. Other viruses utilize diverse EV populations as well. As one of the viruses responsible for viral gastroenteritis, rotavirus releases large plasma membrane-derived EVs [29]. Endosomes are also the site of biogenesis for the exosome-producing EV subgroup. In addition, as early endosomes transform into late endosomes or MVBs, intraluminal vesicles (ILVs) begin to accumulate in the early endosomes via endosomal membrane invagination. These bodies then fuse with lysosomes to promote ILV degradation or with the cell membrane to release ILVs into the extracellular environment. Consequently, these vesicles are typically referred to as exosomes [30], [31].

A series of enzymes called neutral sphingomyelinases (nSMases) change the sphingomyelin found inside lipid rafts into ceramides and also direct the ESCRT-independent process. On account of their interaction, the resultant ceramides create sizable microdomains that trigger the ILVs to develop into MVBs and bloom. With regard to its ability to stop ILV creation and exosome synthesis, GW4869 is thus thought to be a strong nSMase inhibitor [30], [32]. The ESCRT-dependent and -independent pathways are also two primary processes that promote the biogenesis of MVBs. The multi-molecular machinery known as the ESCRT, which consists of numerous proteins capable of interacting with ubiquitinated cargo, also aids in the formation of ILVs. The rat sarcoma virus (RAS), as a small guanosine triphosphate (GTPase) that coordinates diverse cellular activities, such as exosome synthesis and cell proliferation, differentiation, adhesion, and migration appears to be associated with ESCRT pathways. In order for the RAS to function properly, it must be joined to a lipid chain via the activity of enzymes known as farnesyltransferases [33].

Late endosomes, produced by the restricted MVB membrane budding inward, are also the constitutive source of exosomes. Late endosomal membrane invasion consequently generates ILVs in large MVBs. After fusion with the plasma membrane, the majority of ILVs are released into the extracellular environment, where they are known as exosomes [34]. As demonstrated, the ESCRT is essential for ILV development. It is also a complex protein machine composed of four distinct protein ESCRTs (zero through three) that work together to promote vesicle budding, MVB formation, and protein cargo sorting [34], [35], [36].

## 2 Role of EVs in the pathogenesis of ABDs

### 2.1 Chikungunya virus (CHIKV)

The positive-sense, single-stranded (+ss) RNA virus CHIKV belongs to the Togaviridae family of alphaviruses. The CHIKV fever is also known as an acute viral illness that causes excruciating joint pain and is self-limiting. Due to its resurgence with numerous outbreaks and chronic clinical consequences, including persistent arthralgia, infection with CHIKV has become an even more serious global health concern [16]. Post-CHIKV arthritis has been previously defined as a chronic infection characterized by macrophage recruitment and activation, elevations of multiple inflammatory mediators, and the persistence of virus or viral products in the synovial fluid [21]. The CHIKV hides in apoptotic blebs and infects nearby host cells while evading the host’s immune system [16]. Understanding the origin and progression of chronic arthritis after CHIKV infection is crucial, as no specific treatment or vaccination for its prevention has yet been developed.

Researchers have utilized an alphavirus model to comprehend the CHIKV’s replication. Accordingly, the E1 primarily controls fusion in alphaviruses, and it is generally accepted that the conformational remodeling of the E1–E2 envelope heterodimer inside the endosomes, which is favored by low pH, renders them unstable and causes their previously buried fusion peptide to target the membrane briefly. The replication of alphaviruses is subsequently a two-step process. It occurs in the cytoplasm of the host cell and is associated with membrane-like cytoplasmic structures that are copurified with the mitochondria of infected cells. As a result of infection, the structure of the replication complex is altered. Nonstructural proteins (NSPs) regulate the transcription of early-stage negative-stranded and positive-stranded RNAs [37], [38].

Hepatitis virus, HSV, and DENV virus have all been shown to be transmitted by sEVs. Therefore, it has been established that the isolated sEVs are capable of infecting nearby vulnerable cells. According to the findings reported by Le et al. [16], the CHIKV genomic RNA and any immunogenic components of the virus may be contained within the vesicles secreted by CHIKV-infected cells. These EVs could then aid in the production of infectious viruses by other epithelial cells. These results pointed to the potential role of EVs in the spread of CHIKV throughout host organs and their possible contribution to immune evasion-induced chronic arthritis (Table 1 (Tab. 1)).

### 2.2 Zika virus (ZIKV)

The +ssRNA virus, also known as ZIKV, belongs to the family Flaviviridae. The *Aedes* species mosquitoes are also the main vectors of this re-emerging arbovirus, which can spread sexually and vertically from mother to fetus, as evidenced by the transmission of the virus from mother to fetus [18]. Without the assistance of mosquitoes, the ZIKV can also be transmitted via bodily fluids. In addition, the virus has been detected in plasma, cerebrospinal fluid (SCF), amniotic fluid, urine, sperm, vaginal secretions, breast milk, and saliva from infected individuals. Even blood and intrauterine transmission, sexual activity, and lactation have been documented as transmission routes for specific bodily fluids. Since there have been unresolved cases of non-sexual human-to-human transmissions, this mode of transmission has not been completely ruled out, although there is no evidence that the ZIKV can spread through saliva, i.e., during a passionate kiss. Notably, saliva frequently contains ZIKV RNA, which may be advantageous for diagnostic purposes, because RNA concentrations can reach 10^6^ copies per milliliter and be detected for up to 91 days [39], [40].

Recent research has also linked the flavivirus ZIKV to severe congenital disabilities in humans and non-human primates, such as parenchymal brain lesions, microcephaly, ocular lesions, and other developmental anomalies. As the primary interface between the fetus and the mother, the placenta serves as a barrier to prevent the vertical transmission of infections to the fetus [41]. Historically, ZIKV has been regarded as a self-limiting fever illness, and until quite recently, human cases have been documented sporadically, clustering in specific geographic areas. Since the outbreak in Brazil in 2015, the virus has spread throughout the Western Hemisphere, causing an increase in ZIKV infections and even reports of neurological conditions such as Guillain-Barré syndrome (GBS) and infection-induced congenital microcephaly.

Due to the growing global threat posed by Zika-associated congenital syndrome and neuropathies, the scientific community has endeavored to gain a better understanding of the replication and pathogenesis of the virus within the host [7], [18]. Some research suggests that exosomes can cross the placental barrier and transmit signals between the mother and developing fetus, so they have been used to deliver medications to developing fetuses [42]. During the first trimester of pregnancy, when the risk of fetal infection with ZIKV is greatest, the fetal EV circulation in the maternal blood increases. It is plausible that the EVs produced by ZIKV-infected cells may have a similar effect on the host, as it has been demonstrated that virally modified EVs can alter cellular microenvironments and promote virus transmission and pathogenicity [18].

Tabari et al. [43] used next-generation sequencing to examine the EV-derived and intracellular miRNAs and mRNA transcriptomes in ZIKV-infected neural stem cells, given the importance of EVs in the progression of ZIKV infections. They reported that ZIKV infections altered the manner in which host miRNAs, such as miR-4792, were involved in oxidative stress and neurodevelopmental processes as secreted by EVs. In pathological conditions, EVs have also been proposed as biomarkers for viral infection and certain brain disorders. Accordingly, the Trojan-horse theory explains how viral infections can sustain and avoid host immune responses because viral proteins are frequently found within EVs, and viral infections are known to influence EV pathways. However, it is still unclear how EVs and the upstream ceramide pathway cause ZIKV infection [44].

Numerous virus-derived small-interfering RNAs (vsiRNAs) are also produced in mammalian cells to target a wide variety of RNA viruses, including the influenza A virus (IAV), ZIKV, and DENV [24]. According to Martinez-Rojas et al. [45], both small and large EVs from ZIKV-infected *Aedes* mosquito cells were able to alter the host cell responses, which may have been associated with the pathogenic pathways leading to the development of severe disease types. Table 1 (Tab. 1) presents findings on the role of EVs in the pathogenesis of ZIKV based on an analysis of the papers recruited for this review: four studies demonstrated the role of EVs in preventing viral pathogenesis.

Malamud et al. [46], for instance, reported that human saliva contains antimicrobial and antiviral activity. Analysis of the effect of human saliva on the ZIKV infection revealed that saliva could inhibit the infection by preventing ZIKV attachment to target cells. Saliva-abundant EVs that competed with ZIKV for cellular interactions were responsible for this, representing a novel antiviral defense mechanism. In addition, Zou et al. [42] observed that the interferon (IFN)-induced transmembrane protein 3 (IFITM3) was an intrinsic cellular factor that inhibited infections caused by a number of influential viral pathogens. IFITM3 was predominantly localized on the endolysosomal membrane and inhibited early viral replication. Blocking the fusion of viruses with endolysosomal membranes could further inhibit virus entry, thereby reducing infection.

Similarly, the IFITM3 has been shown to prevent ZIKV infection, which is notable and effective. It is also believed to play a role in the fetus’ defense against infections. Because it is strongly expressed in the placenta and increases in expression throughout pregnancy, it is probably well-tolerated and safe during fetal development [42]. Wang et al. similarly demonstrated that EVs from sperm are potent inhibitors of the ZIKV infection at physiological concentrations, which may explain why sexual transmission of ZIKV is relatively uncommon. Using the same volume of seminal plasma devoid of sEV also resulted in significantly less inhibition, indicating that the inhibitory effect of semen on the ZIKV was related to the EV proportion [40].

According to Vojtech et al., human sperm contains trillions of EVs containing bioactive miRNAs, proteins, and lipids, which account for the size of sexually transmitted viruses. Although it has been shown that sEVs can reduce the infectiousness of HIV and ZIKV, it is not yet known whether these vesicles can influence future immunological responses [19]. According to previous research, it is crucial to identify the trafficking and function of fetomaternal exosomes during pregnancy. It was confirmed that IFITM3-expos transported the IFITM3 molecules and inhibited the ZIKV infection in both mother and fetus using cell culture and pregnant-mouse models. It has been suggested that the exogenous IFITM3 supplied by EVs could be a promising treatment for preventing prenatal ZIKV infection [42].

In contrast to the time when EVs played a crucial role in preventing the spread of the virus, some research indicated that they could gradually reduce the virus’ infectiousness. EVs and exosomes may play a role in the pathophysiology of the ZIKV infection in human hosts, as confirmed by Mao et al. Exosomes and EVs containing viral RNA and the ZIKV E protein could be released by mosquito cells (C6/36) and transmitted from an infected mosquito to its hosts due to the presence of the ZIKV in the *Aedes* species. These ZIKV-causing Zika C6/36 EVs/exosomes were able to infect human monocytes and vascular endothelial cells as their primary targets [47]. In this context, Li et al. reported that ZIKV-produced nucleic acids and proteins may be present in exosomes to facilitate infection. Exosomes may modulate the expression levels of nSMase two in cortical neurons to facilitate ZIKV transmission (see Table 1 (Tab. 1)) [48].

In addition, ZIKV poses a threat, especially to pregnant women, because neither a vaccine nor a treatment is available [39]. High levels of ZIKV RNA, up to 10^9^ copies per milliliter of infected men’s sperm, have been found to persist for up to nine months following the onset of symptoms [40]. Additionally, the EVs carrying the miR517-3p, miR16B-5p, and miR512 exhibited potent antiviral activity in the recipient cells. It has been observed that EVs produced from the sperm of ZIKV-infected patients can inhibit this condition [49].

By transferring viral RNA to naive recipient cells, EVs originating from ZIKV- or DENV-infected cells contribute to the spread of the viruses [50]. Accordingly, additional research has revealed that ZIKV-infected macrophage-derived EVs are responsible for the activation of the NLR family pyrin domain containing 3 (NLRP3) inflammasome, the hyperactivity of caspase-1 (CASP1), and the secretion of IL-1, all of which result in the induction of innate immunity in certain organs of the host. Furthermore, once the ZIKV enters the central nervous system (CNS), EVs mediate the ZIKV infection and spread throughout neural cells. Moreover, the ZIKV stimulates the production of EVs in infected cells and enters astrocytes with significantly greater efficiency than other types of brain cells [51], [52].

### 2.3 DENV

The DENV, the most significant mosquito-borne Flaviviridae virus, has imposed significant socioeconomic and health-related burdens worldwide [53]. Similarly, it is responsible for a greater burden of human disease than any other arbovirus, causing an estimated 10,000 deaths and 100 million symptomatic infections annually in more than 125 countries [25]. According to the World Health Organization (WHO), approximately half of the world’s population is currently at risk of DENV infection [26], [54]. Four serotypes of the DENV are the most important arboviruses causing human disease (namely, serotypes 1–4). These viruses are uncommon in the reservoir hosts that are humans [9]. The fifth edition, DENV-5, has thus been identified as the most recent [55].

In contrast to the other four serotypes, which follow the human cycle, DENV-5 follows the sylvatic cycle, in which humans serve as the final hosts. More than 50% of the world’s population is projected to be affected by 2050 [28]. Only 10% of febrile illness cases are reported to the WHO, resulting in an estimated 400 million annual human infections [7]. In tropical and subtropical nations, *Aedes* mosquitoes are primarily responsible for the endemic and epidemic transmission cycles [53]. In addition, non-human primates serve as reservoir hosts in the DENV transmission cycle, as demonstrated by studies of virus ecology in sylvatic habitats in West Africa and Malaysia [9].

While the majority of DENV infections in humans are asymptomatic, a small proportion of cases exhibit clinical symptoms, ranging from a self-limiting mild flu-like illness known as dengue fever (DF), which resolves without complications, to more severe conditions [53]. Thrombocytopenia and vascular leakage are clinical characteristics of these severe dengue infections, which include bleeding, e.g., dengue hemorrhagic fever (DHF), and multi-organ involvement, such as dengue shock syndrome. In the context of hemorrhagic complications, patient mortality rates can therefore increase by up to 20% [26]. The exosomes that cause infection by DENV have distinct compositions. Viruses that infect arthropod cells generate a full-length DENV genome in addition to other proteins. It has been discovered that the exosomes produced by DENV-infected cells are larger than those produced by healthy, non-infected cells, probably so that exosomes can contain the complete viral genome and proteins [28].

Recent research has shown that tick and mosquito sEVs, including exosomes, facilitate the transfer of arboviruses between arthropod and mammalian cells as well as between arthropod cells. Table 1 (Tab. 1) contains a summary of the results. Mishra et al. demonstrated that these secreted EVs positively support the pathogen’s life cycle and effective pathogenesis [56]. However, they can function as a messenger for the activation of antiviral mechanisms in the host. Exosomes produced by mammalian cells were present during the DENV infection, according to Reyes-Ruiz et al [17]. They were also involved in the transmission of antiviral response mediators between cells.

There have been recent reports on the isolation and characterization of EVs from DENV-infected mosquito cells. According to Vora et al., DENV2/DENV3-infected cells secreted EVs ranging in size from 30 to 250 nm that contained infectious viral RNA and proteins. The mosquito and mammalian cells may be infected further by the DENV2 genome found in its entirety in EVs [28], [57] (Table 1 (Tab. 1)).

According to previous research, human low-density lipoprotein (LDL) can enter mosquito cells via clathrin-mediated endocytosis (CME) and prevent both in-vitro and *in-vivo* flavivirus infection. Furthermore, LDL protects against flavivirus infection early in the virus’s life cycle. LDL molecules with a diameter of 22–27.5 nm have been found in EV preparations, such as exosomes, MVs, and ABs [58]. Their transmembrane shape is thus a hurdle for cross-cell transport, according to Zou et al. Exosomes may be further exploited as a delivery system for the IFITM3 to treat viral infection. In this respect, a recent study found that exosomes could intercellularly transfer the IFITM3 during the DENV infection and convey its antiviral impact from the virus-infected cells to the non-infected ones [42].

In addition to acting as a complement, the DENV can cause platelets to produce EVs (also known as microparticles), which aid neutrophil extracellular trap (NET) formation and the DENV-induced inflammatory responses. All cell types are capable of producing EVs. Nonetheless, platelets are the primary source of EVs in circulating plasma sera [59]. There is no specific treatment for DENV, and there are few remaining vaccine options. To improve the prognosis of patients, therefore, it is necessary to develop novel anti-infection strategies, such as identifying biomarkers to pinpoint cases more likely to develop a severe illness and identifying prospective treatment targets [26].

In this context, Martins et al. [60] reported that vesicles from infected cells could carry IFN-stimulated gene mRNAs, and confirmed that vesicles from IFN-treated cells could have a protective effect against infection, effectively blocking it in some neighboring cells. These results suggested that the signals involved in the IFN-mediated response could be carried by EVs and protect against DENV infection as immune cells. Consequently, the IFN-induced transmembrane proteins (IFITMs) comprise a group of proteins, namely IFITM1, IFITM2, and IFITM3. The given compounds possess significant antiviral properties and function as effector molecules, inhibiting the entry of diverse enveloped virus types and influencing their cellular tropism independently of receptor expression. Adopting a cellular model of DENV infection, one study concluded that exosomal IFITM3 could effectively prevent virus entry under both baseline and IFN-induced conditions [28].

Moreover, Vora et al. [1] concluded that the discovery of a glycoprotein, Tsp29Fb, containing the tetraspanin domain which was hypothesized to be the ortholog of human CD63 (as a marker for mammalian exosomes/EVs), provided evidence for conservation in intercellular communication mediated by EVs. Therefore, it was proposed that inhibiting this communication could be a fundamental therapeutic strategy for preventing DENV2 transmission from arthropods to humans. 

Freitas et al. [58] also observed that EVs might be capable of aggregating DENV and facilitating its entry via phagocytosis. EVs may also induce DENV fusion, inhibiting its fusion with the endosomal membrane and accelerating its degradation in the lysosome. In contrast to previous research, it was observed that exosomes derived from DENV-infected mosquito cells contained infectious DENV RNA and proteins. This indicated that such exosomes possessed the potential to serve as the agents of DENV transmission from mosquito to mammalian cells [61]. The presence of positive- and negative-stranded viral RNA, NS1, and E proteins in exosomes derived from tick cells infected with LGTV or mosquito cells infected with DENV2/DENV3 suggested that arthropod-borne viruses or arboviruses could use exosomes as mediators for intercellular communication between infected and uninfected recipient cells [62].

As reported in a separate study, the induction of NETs by DENV led to a significant increase in vascular permeability compared to control groups. In addition, a decrease in NET formation and an increase in vascular permeability were observed in mice as well as in-vitro upon combining the blockade of C-type lectin-like receptor 5A (CLEC5A) and toll-like receptor 2 (TLR2) with the administration of nuclease to digest the NETs [63]. Recent research has demonstrated that mosquito sEVs play a crucial role in the spread of DENV. In this regard, Sung et al. [59] demonstrated that NET deposition in the spleen may significantly contribute to the increased vascular permeability and subsequent lethality caused by DENV (specifically DENV-2 subtype) in mice. Experiments conducted in-vitro indicated that DENV infection of human neutrophils could result in the release of NETs, which have a detrimental effect on endothelial cells. In addition, the intercellular communication mechanisms underlying the observed phenomenon were elucidated, and the potential for therapeutic interventions to treat this tropical disease was discussed [63].

### 2.4 West Nile virus (WNV)

WNV is a member of the JEV serogroup within the flavivirus genus and Flaviviridae family. It is a small virus with an envelope that contains a +ssRNA genome. Thus, the viral RNA is capped but lacks a polyadenylated tail. In addition to encoding three structural and seven nonstructural proteins, the viral genomic RNA is also responsible for encoding noncoding (nc) RNA. Additionally, the entity displays a pervasive distribution pattern throughout temperate and tropical regions worldwide.

The WNV was discovered in Uganda in 1937 and has resided exclusively in the Old World until recently. During the 1950s, the onset of a febrile human disease caused by the WNV was first documented in Israel. In light of this, the WNV and JEV share numerous ecological characteristics. The WNV’s endemic transmission mechanism involves the virus’ transmission between avian species via *Culex* spp. The pathogen is also transmissible to human and equine terminal hosts [9], [64].

As with the JEV, birds are effective at transmitting the WNV because they carry the virus in their blood for several days. This makes it possible for migratory birds to spread the virus over vast distances, approximately 1 out of every 150 cases of WNV infection results in animal illness. In the past, WNV was primarily associated with fever and rarely caused brain inflammation. This disease appeared in New York for the first time in 1999 and is now widespread throughout the United States. It can infect numerous animals, including 49 species of mosquitoes, 225 species of birds, and ticks. Notably, the WNV has not yet been transmitted to other domesticated animals, including horses, cows, llamas, cats, dogs, wolves, and sheep. The WNV can cause additional illness in humans, but it may not be able to spread from person to person (Table 1 (Tab. 1)) [9].

In some cases, particularly among older adults and immunocompromised individuals, infection with the West Nile virus (WNV) may result in fatal outcomes or significant long-term neurological sequelae, such as tandem gait impairment, hearing loss, abnormal reflexes, and muscle weakness [65]. Some viruses, such as the Ebola virus VP24, VP40, and NP, the influenza virus NP, Crimean-Congo hemorrhagic fever NP, WNV NS3, and HCV NS3, have now been shown to be susceptible to exosome vaccines in-vivo. This technology must now be evaluated in clinical settings [66].

Notably, EVs facilitate the transfer of chemicals between cells and can even transmit antiviral activity. Therkelsen used next-generation sequencing to describe the RNA cargo of the EVs produced in response to WNV infection and IFN-α treatment in 2019, and found that short RNAs from EVs secreted by WNV-infected cells can stimulate the expression of antiviral genes [67]. A comparative analysis of the RNA-seq data for RNAs revealed that infection significantly altered the levels of host miRNAs, small noncoding (snc) RNAs, and mRNAs in EVs. In addition, it was observed that both IFN-dependent and -independent pathways regulate the sorting of RNA into EVs following infection. 

Once RNAs were functionally categorized, it was demonstrated that they were associated with viral activities and antiviral pathways and that they were variably integrated into EVs following infection and IFN treatment [64]. Considering their ability to transport infectious RNA and viral proteins, EVs may play a crucial role in the propagation of flaviviruses (Table 1 (Tab. 1)). These vesicles initially serve as viral propagation substitutes and then facilitate immune evasion [67]. Various studies have reported the protective role of endosomal alkalinization in preventing infection with WNV and other flaviviruses, including ZIKV and yellow fever virus, by controlling the fusion of the virion endosomal membrane.

Therefore, it is intriguing to hypothesize that the paracrine transport of sodium hydrogen exchanger (NHE) mRNA by EVs from WNV-infected cells alkalinizes endosomal pH, thereby preventing viral entry into bystander cells. According to one study [64], the WNV infection altered the levels of specific host miRNAs, sncRNAs, and mRNAs that were incorporated into EVs. The IFN-based treatment also altered the miRNA and mRNA profiles of EVs, but its effect on sncRNAs was less pronounced [64].

### 2.5 Tick-borne encephalitis virus (TBEV)

The considerable morbidity and mortality caused by vector-borne diseases are currently a significant concern. The rickettsial pathogens (such as *Rickettsia rickettsia* and *Anaplasma phagocytophilum*), *Francisella tularensis*, a bacterium that causes tularemia, and the recently discovered Powassan virus (POWV) are among the severe diseases transmitted to humans and animals by the medically important *Ixodes scapularis* ticks [42]. TBEV possesses a +ssRNA genome [51]. TBEV virion size is also 30–50 nm, and it is the most significant tick-borne infectious disease from a medical standpoint. The host’s endoplasmic reticulum (ER) and Golgi complex are the sites of TBEV assembly and viral particle maturation, where the viral surface protein E is incorporated into the virion. After virion assembly, the host proteins can be incorporated into the virion [68]. To the best of the author’s knowledge, despite an exhaustive search, no paper explicitly addressing the impact of EV on this virus could be located.

### 2.6 Japanese encephalitis virus (JEV)

As a member of the Flaviviridae family, the JEV is a +ssRNA linear virus with an approximate 11 kb genome. This virus is the most prevalent cause of viral encephalitis in Asia and India and is capable of infecting brain cells [69]. Although the JEV has been known to cause disease since the 1870s, it was not identified until 1935 in the brain of an encephalitis victim in Tokyo, Japan. The JEV is the leading cause of pandemic encephalitis worldwide. The virus primarily affects India, Korea, China, Southeast Asia, and Indonesia, where it causes epidemics of pediatric encephalitis. Additionally, more than two billion people are at risk of infection due to its widespread geographic distribution and case fatality rates that frequently exceed 20%. Approximately 50,000 infections occur annually, with 15,000 cases resulting in death; more importantly, up to 50% of those who survive the illness experience neurological side effects that persist for months to years [9], [70].

*Culex tritaeniorhynchus* mosquitoes are the primary JEV vectors, and they are most likely to isolate the virus from humans and horses in late July. In Malaysia, both *Culex glides* and *Culex tritaeniorhynchus* are significant vectors. In addition to *Aedes*, other mosquito vectors include *Anopheles*, *Mansonia*, and *Armigeres* [9]. This virus primarily infects children and adults, and 30 to 50% of survivors experience long-term neurological effects [69]. The symptoms of JEV infection in humans can range from a mild fever to severe hemorrhagic and encephalitic symptoms or death [34], despite the fact that the vast majority of human cases of JEV infection are asymptomatic. Neuroinflammation is another characteristic of JEV infection. It is a neurotropic virus that affects children between the ages of 1 and 5 and causes memory loss, persistent neuronal damage, and motor impairments [48].

As a single-chain, single-pass transmembrane glycoprotein that regulates lipid metabolism and lipoprotein trafficking, the LDL receptor (LDLR) has been reported to bind to the JEV-E and play a role in JEV entry into host cells, indicating that it may serve as a cellular receptor for this virus. In addition, berberine – a bis-benzylisoquinoline alkaloid derived from Berberis shrubs and trees which is a well-known inhibitor of calcium channels or signaling – significantly decreased the LDLR at the plasma membrane, thereby rendering cells immune to the JEV infection. Consequently, EVs were able to secrete more LDLR, and their plasma membrane levels decreased, preventing JEV from entering host cells [6].

In recent years, it has been suggested that JEV infection can significantly alter the intracellular and extracellular miRNA profiles in the brain and that numerous miRNAs may be essential for regulating viral replication and neuroinflammation. The attenuated SA-14-14 JEV vaccine is now widely available and has variable effectiveness. For the treatment of neuroinflammation caused by the JEV, there are currently no effective anti-JEV medications or techniques, and only life-sustaining treatments are administered. Therefore, additional research on alternative JEV treatment strategies is still required [69].

### 2.7 Tick-borne langat virus (LGTV)

The tick-borne LGTV, a model pathogen closely related to the TBEV, can make extensive use of arthropod exosomes in order to transmit viral RNA and proteins to human skin keratinocytes and blood endothelial cells. Due to the substantial genomic similarity between the LGTV and the TBEV, it is used as a BSL2 model pathogen to investigate the pathophysiology of the latter. These arthropod-transmitted +ssRNA flaviviruses have transmission mechanisms that are poorly understood at present [71], [72].

Exosomes produced by arthropod, mouse, and human cells contain both positive and negative strands of LGTV RNA and viral envelope protein. The discovery of nonstructural 1 (NS1) protein in the exosomes of arthropods and neurons provides additional evidence that exosomes carry viral proteins. Large quantities of exosomes from neurons infected with both WNV and LGTV have been identified. *Ixodes scapularis*, a medically significant vector tick, secreted exosomes from its cells which mediated the transfer of tick-borne LGTV RNA and proteins from arthropod to human, as demonstrated in one study [72]. Exosomes produced by tick cells infected with LGTV were discovered to be capable of migrating and affecting non-infected human skin keratinocytes and human vascular endothelial cells. 

Liao et al. similarly demonstrated that the exosomes produced by tick cells infected with LGTV contained infectious virus RNA and protein, thereby facilitating the transmission of LGTV from tick to vertebrate host cells [4], [8]. According to Wower et al. [73], the tick-borne LGTV, a flavivirus similar to the TBEV, could be another example of RNA transport facilitated by exosomes. Table 1 (Tab. 1) demonstrates that the exosomes produced by tick and human cells infected with the LGTV transmit infectious viral RNA and nonstructural proteins to unaffected cells [64]. Overall, it has been hypothesized that exosomes not only mediate the spread of the arthropod-borne flavivirus RNA and proteins to the vertebrate host but also help spread these infectious RNA and proteins throughout the vertebrate host by allowing the crossing of the BBB cells and facilitating neuroinvasion and neuropathogenesis in the CNS [4], [62].

Infected murine brain endothelial barrier cells produce exosomes that transmit infectious RNA and proteins to murine neuro 2A (N2a) cells. Neuropathogenesis and neuronal death result from neuronal cells infected with LGTV releasing exosomes that spread the virus deeper into the brain [49].

### 2.8 Usutu virus (USUV)

Since the USUV has spread extensively outside of Africa, particularly in Europe, it has attracted the attention of scientists in recent years as one of the potential emerging viruses [74]. The USUV is a member of the virus family Flaviviridae, which is primarily transmitted by mosquitoes. The USUV has an icosahedral envelope with a diameter between 40 and 60 nm. Its genome consists of an 11 kb +ssRNA with a 5' cap structure that codes for a polyprotein with 3,434 amino acids. In 1959, the USUV was first identified in a *Culex* mosquito in South Africa. It was then isolated by inoculating young mice intracerebrally. It is typically sustained by an enzootic cycle in which ornithophilic mosquitoes, such as *Culex* *pipes*, serve as vectors and birds (primarily Passeriformes and Strigiformes) serve as amplifying hosts [75]. Accidental hosts include humans as well as animals such as dogs, horses, rats, and wild boars. Consequently, significant WNV epizootics with avifaunal effects were observed in Europe in 2016 and 2018 [76].

The majority of ZIKV and USUV infections are asymptomatic, but they can cause conditions ranging from mild febrile illness to severe neurological complications [10]. Exosomes and MVs also play essential roles in the pathogenesis and life cycle of numerous RNA viruses, such as the transfer of the full-length genomic RNA of the hepatitis C and hepatitis G viruses, the HIV-derived miRNAs, and the antiviral protein APOBEC3G [64]. It has been reported that tick cells produce exosomes that carry tick-borne LGTV, raising the possibility that other tick-borne flaviviruses, such as TBEV and POWV, may also exploit this novel method of arthropod transmission [4]. In addition, breast milk includes EVs and glycosaminoglycans (GAGs) that prevent the ZIKV and USUV from attaching to cells [77].

## 3 GW4869 as an inhibiting pharmacological agent to stop exosome generation

The nSMase enzyme is essential for exosome production and release. It then prevents the release of sEVs by producing lipid ceramides by hydrolyzing the membrane lipid sphingomyelin [4], [34]. Additionally, GW4869 is a cell-permeable, selective inhibitor of this enzyme. Following treatment with GW4869, the amount of LGTV in exosomes produced by arthropod and mammalian cells can be altered, thereby preventing exosome release. Exosomes from infected brain-microvascular endothelial cells, which comprise the BBB, have also increased LGTV RNA and protein transmission, barrier crossing, and neuronal cell infection in transwell-migration assays. The effect of the GW4869-based treatment on LGTV replication, loading, and transmigration from one cell type to another suggests that the nSMase or pathways associated with it may be utilized by the LGTV or other tick-borne flaviviruses to package into exosomes [4].

Additionally, the exosomes from arthropods and neurons treated with GW4869 inhibit the production of viral RNA and proteins and the spread of flaviviruses to unprepared recipient host cells. The mechanisms by which GW4869 affects viral transmission are unknown [5].

The ZIKV infection also increases the expression and activity of nSMase2. GW4869 treatment and nSMase2 inhibition have decreased the frequency and burden of exosome-mediated viral transmission. Accordingly, it has been hypothesized that exosomes may aid in the spread of ZIKV, although the underlying mechanisms remain unknown [51]. According to the effects of GW4869 on the LGTV infection in N2a cells, the inhibition of exosomes before or after infection would have similar effects on LGTV loads and transmission [78]. GW4869, an enzyme that regulates the synthesis and release of exosomes, has been shown to decrease the LGTV loads in exosomes by inhibiting the transmission of the LGTV RNA and proteins in both arthropod and vertebrate host cells [51]. The ZIKV propagation and release in human fetal astrocytes was inhibited further by the GW4869 suppression of EV release [44]. In primary cultures of murine cortical neurons, where an increase in exosome formation was observed after ZIKV infection, similar results were also observed [51]. 

## 4 Conclusion and outlook

Typically, arboviruses are transmitted by arthropods such as mosquitoes, ticks, and sandflies. EVs are also known as small membrane-bound vesicles that are released by cells and play a vital role in intercellular communication. Exosomes have been suggested as potential missing links in the emergence of DHF in the context of dengue infection. Using the EVs produced by ZIKV-infected endothelial cells that contain ZIKV components, Zika can further compromise the BBB [7]. Moreover, the EVs produced by virus-infected cells can prevent the spread of infectious diseases [50]. Exosomes produced by tick neuronal cells have been shown to contain both positive- and negative-sense RNA strands, suggesting that exosomes facilitate the transfer of both types of RNA genomes [4]. EVs can aid the host cell in suppressing virus infection by inducing cytokine release and antiviral responses [24].

EVs, including exosomes, secreted by medically significant arthropods such as ticks and mosquitoes, further facilitate the transmission of flavivirus RNA and proteins to vertebrate cells [78]. Understanding the role of EVs in RNA virus infection has progressed substantially up to this point. Typically, they use three significant mechanisms to facilitate viral transmission. EVs initially transport viral receptors to make recipient cells more susceptible to viral infection. Second, they transmit infectious viral particles directly to expedite the spread of the virus. Thirdly, EVs serve as a barrier between viruses and immune cells that could otherwise degrade or identify them.

Consequently, EVs represent a potential therapeutic target for preventing the infection and transmission of RNA viruses. To support the potential viability and efficacy of this method, rigorous clinical trials and extensive in-vitro and *in-vivo* investigations are required [51]. This review demonstrated for the first time that exosomes are the new vectors for flaviviruses that are transmitted by arthropods and can infect a wide variety of vertebrate hosts, including humans.

EVs may have contradictory functions during viral infections and pathologies. This review provides a list of clinically significant viruses that have been observed to interact with EVs, as well as the pathophysiology and implications of the viral infection caused by EVs. Notably, these vesicles are essential for intracellular communication and may also represent a novel antiviral defense mechanism. Viruses utilize the EV biogenesis pathway to propagate viral infection, replication, and disease dissemination. Viruses also utilize EVs to enhance their pathogenesis and regulate antiviral immune responses. Overall, EVs are strongly associated with the infection of Flaviviridae viruses, presumably by transporting viral regulatory components and regulating innate immune responses. It has been established that EVs, particularly RNA viruses, facilitate the spread and human infection of other viruses. 

Previous research has demonstrated that flaviviruses, such as the tick-borne LGTV or the mosquito-borne DENV, ZIKV, or WNV, are transmitted from the vector to the vertebrate host via arthropod exosomes [5]. This RNA virus also induces tick cells to release exosomes, which are ingested by neural cells. It is fascinating to note that infected neural cells can release exosomes containing LGTV RNA. These results are consistent with the hypothesis that EVs can transport both large and small RNA molecules. Consequently, future research is anticipated to identify the extracellular viral lncRNAs that are essential for pathogenesis [73]. These findings suggest that targeted inhibition with modified exosomes can significantly contribute to the development of novel treatments for vector-borne diseases. It is therefore recommended to place a strong emphasis on the use of engineered exosomes as a strategy for combating vector-borne diseases through vaccine development. It is preferable to emphasize the use of vaccines containing protein antigens expressed on EV membranes, which are highly immunogenic against flavivirus infections such as dengue and ZIKV [79].

The application of sEVs in ABD management is still in its infancy, and their use and development face significant barriers. First, the mechanistic aspects of how these vesicles function during insect-pathogen interactions must be better understood. Second, scalability and yield optimization must be addressed for the effective implementation of EV-based therapeutics produced on an industrial scale. In conclusion, it is recommended to consider the crucial role of sEV-mediated transfer in the spread of ABDs by moving pathogenic agents between cells within vectors, leading to subsequent transmissions to hosts. However, these vesicles provide potential targets for the development of novel therapies that inhibit the replication of pathogens or reduce the populations of arthropod vectors. Future research in this area should, therefore, utilize advanced imaging techniques, such as high-resolution microscopy (HRM), to shed light on how these vesicles function within host-vector systems, as well as develop cost-effective methods to produce large quantities required for successful implementation on a large scale.

## Notes

### Authors’ ORCIDs

- Owliaee I: 0000-0002-9695-4938
- Khaledian M: 0000-0002-8123-7856
- Shojaeian A: 0000-0002-1166-385X

### Competing interests

The authors declare that they have no competing interests.

## Figures and Tables

**Table 1:**
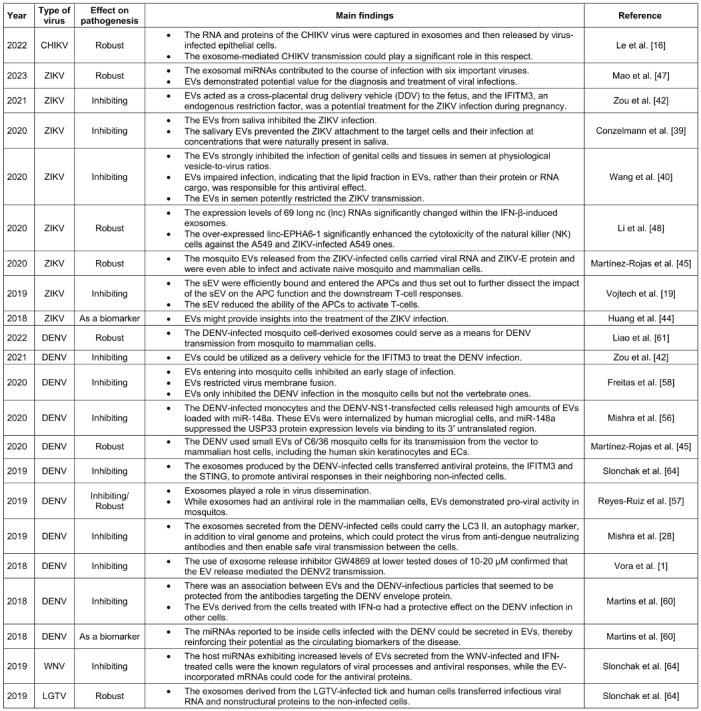
The role of exosomes in inhibiting and enhancing ABD
